# Predicting missed health care visits during the COVID-19 pandemic using machine learning methods: evidence from 55,500 individuals from 28 European countries

**DOI:** 10.1186/s12913-023-09473-w

**Published:** 2023-05-25

**Authors:** Anna Reuter, Šime Smolić, Till Bärnighausen, Nikkil Sudharsanan

**Affiliations:** 1 Heidelberg Institute of Global Health, Heidelberg University, Heidelberg, Germany; 2 Department of Economics, University of Göttingen, Göttingen, Germany; 3 Faculty of Economics and Business, University of Zagreb, Zagreb, Croatia; 4 Professorship of Behavioral Science for Disease Prevention and Health Care, Technical University of Munich, Munich, Germany

**Keywords:** Missed care, COVID-19, Prediction, Machine learning, Europe

## Abstract

**Background:**

Pandemics such as the COVID-19 pandemic and other severe health care disruptions endanger individuals to miss essential care. Machine learning models that predict which patients are at greatest risk of missing care visits can help health administrators prioritize retentions efforts towards patients with the most need. Such approaches may be especially useful for efficiently targeting interventions for health systems overburdened during states of emergency.

**Methods:**

We use data on missed health care visits from over 55,500 respondents of the Survey of Health, Ageing and Retirement in Europe (SHARE) COVID-19 surveys (June – August 2020 and June – August 2021) with longitudinal data from waves 1–8 (April 2004 – March 2020). We compare the performance of four machine learning algorithms (stepwise selection, lasso, random forest, and neural networks) to predict missed health care visits during the first COVID-19 survey based on common patient characteristics available to most health care providers. We test the prediction accuracy, sensitivity, and specificity of the selected models for the first COVID-19 survey by employing 5-fold cross-validation, and test the out-of-sample performance of the models by applying them to the data from the second COVID-19 survey.

**Results:**

Within our sample, 15.5% of the respondents reported any missed essential health care visit due to the COVID-19 pandemic. All four machine learning methods perform similarly in their predictive power. All models have an area under the curve (AUC) of around 0.61, outperforming random prediction. This performance is sustained for data from the second COVID-19 wave one year later, with an AUC of 0.59 for men and 0.61 for women. When classifying all men (women) with a predicted risk of 0.135 (0.170) or higher as being at risk of missing care, the neural network model correctly identifies 59% (58%) of the individuals with missed care visits, and 57% (58%) of the individuals without missed care visits. As the sensitivity and specificity of the models are strongly related to the risk threshold used to classify individuals, the models can be calibrated depending on users’ resource constraints and targeting approach.

**Conclusions:**

Pandemics such as COVID-19 require rapid and efficient responses to reduce disruptions in health care. Based on characteristics available to health administrators or insurance providers, simple machine learning algorithms can be used to efficiently target efforts to reduce missed essential care.

**Supplementary Information:**

The online version contains supplementary material available at 10.1186/s12913-023-09473-w.

## Background

Disruptions to the delivery of health care can have serious negative effects on individuals’ health. Pandemics are a particular threat to the delivery of health care, as they affect whole health care systems, leaving individuals without any alternative health care options. During the first COVID-19 wave in Europe, primary care visits, hospital admissions, and emergency department visits all declined substantially, already before lockdowns were imposed [1–4]. Even hospital admissions for serious acute health conditions such as heart failure and myocardial infarction decreased considerably [5, 6]. These care disruptions are thought to be a main reason why cardiovascular disease mortality increased in the early stages of the pandemic [7, 8]. Disruptions to essential care may also have longer term effects that persist beyond the pandemic [9]. In Germany, for example, diagnoses of diabetes, dementia, depression and stroke decreased to a larger extent than the number of physician consultations [4], indicating that those in need of preventive care delayed essential health care visits.

Individuals at risk for care disruptions need to be efficiently contacted and reconnected to health services to prevent them from forgoing or delaying essential care. Hospital administrators and health insurance providers are particularly well placed for this role as they can contact patients quickly and at scale. However, they face two important challenges. First, targeting efforts need to be accurate and straightforward without requiring substantial additional resources to not overwhelm the limited capacities of health systems already under pressure. The health system strain created by the COVID-19 pandemic, for example, makes contacting all patients challenging; instead, health administrators and insurance providers need a way of filtering patients by their risk of care disruptions and contacting those with the greatest needs. A second challenge is that while health administrators and insurance providers hold information on health care visits, they usually cannot observe whether they were postponed or cancelled and why. Building prediction models using existing large-scale survey data has the potential to address both of these gaps. Survey data contain information on patients and whether they missed visits. This allows for building models that predict the risk of missed visits using the patient characteristics available to health administrators and insurance providers. These models can then be applied by health administrators and insurance providers to existing patient information to predict each patient’s risk of missed visits, and based on this predicted risk, target efforts to those who would benefit the most.

In this study, we employ four popular machine learning algorithms on data from multiple waves of the Survey of Health, Ageing and Retirement in Europe (SHARE). We build and evaluate models that use common patient characteristics to predict missed health care visits due to COVID-19. First, we build the models based on data from the first SHARE COVID-19 survey in summer 2020 and evaluate them using cross-validation. Second, we assess the external validity and long-term suitability of these models by applying them to data from the second SHARE COVID-19 survey in summer 2021 and assessing how well the models predict missed visits in this future wave. In addition, we analyze algorithmic fairness to indicate potential gaps in targeting. Our findings have immediate and long-term relevance. The models we present here can be used by health administrators and insurance providers to target individuals with efforts to encourage continuity of care for current and future waves of the COVID-19 as well as other future health care disruptions.

## Methods

### Sample

We use data from the SHARE waves 1–8 and the first and second wave of the COVID-19 survey [10–19]. The SHARE panel includes health and socioeconomic information on respondents aged 50 or older and their partners, and covers the European Union (except Ireland), Switzerland and Israel [10]. While the in-person data collection for wave 8 had to be stopped due to the outbreak of COVID-19 in March 2020, computer-assisted telephone interviews were conducted between June and August 2020 for a special survey on COVID-19, covering 57,559 individuals [20]. One year later, between June and August 2021, these individuals were contacted again for a second COVID-19 survey, in which 49,253 individuals participated.

The SHARE study was approved by the Ethics Committee at the University of Mannheim (waves 1-4) and by the Ethics Council of the Max‐Planck‐Society (waves 5‐8). Additionally, country-specific ethics committees or institutional review boards approved implementations of SHARE in the participating countries. All study participants provided informed consent.

### Missed health care visits

Our primary outcome is whether an individual did not attend an essential health care visit. The SHARE COVID-19 questionnaire asked individuals whether they experienced three types of missed health care visits due to COVID-19: (1) forgone medical treatment due to fear of becoming infected; (2) postponement of scheduled treatments by the health provider due to COVID-19; (3) denied appointment since the outbreak of the pandemic. Our main analyses consider any reported missed health care visit; secondarily, we also examine the different reasons for missed health care visits separately. We excluded missed visits to dentists and specialists (one mutual answer category in the questionnaire) as we deem it likely that missed visits in this category are mainly driven by postponed dentist check-ups and thus do not constitute essential care. We include missed visits to general practitioners, planned medical treatments or operations, physiotherapy, psychotherapy, and rehabilitation.

### Predictors

We focus our analysis on possible predictors of missed health care visits that health insurance providers and hospital administrators are likely to have access to. This includes age, sex, education, latest reported employment status, past diagnoses, and past medication use. We defined past diagnoses and medication as the respondent stating in any of the past SHARE waves that a certain condition was (ever) diagnosed/a certain medication was taken. We dropped diagnoses and medications that were asked for irregularly across SHARE waves (asthma, arthritis, osteoporosis, benign tumor, chronic kidney disease, drugs for asthma, drugs for osteoporosis, drugs for suppressing inflammation) to circumvent sample selection problems. Except for age, all predictors are categorical. Sex is measured with an indicator for being female (the data distinguishes between male and female), education with six distinct categories (none, primary, lower secondary, upper secondary, post-secondary, and tertiary education), and employment with six categories as well (retired, working, unemployed, disabled, homemaker, other).

### Missingness

**Fig. 1:**
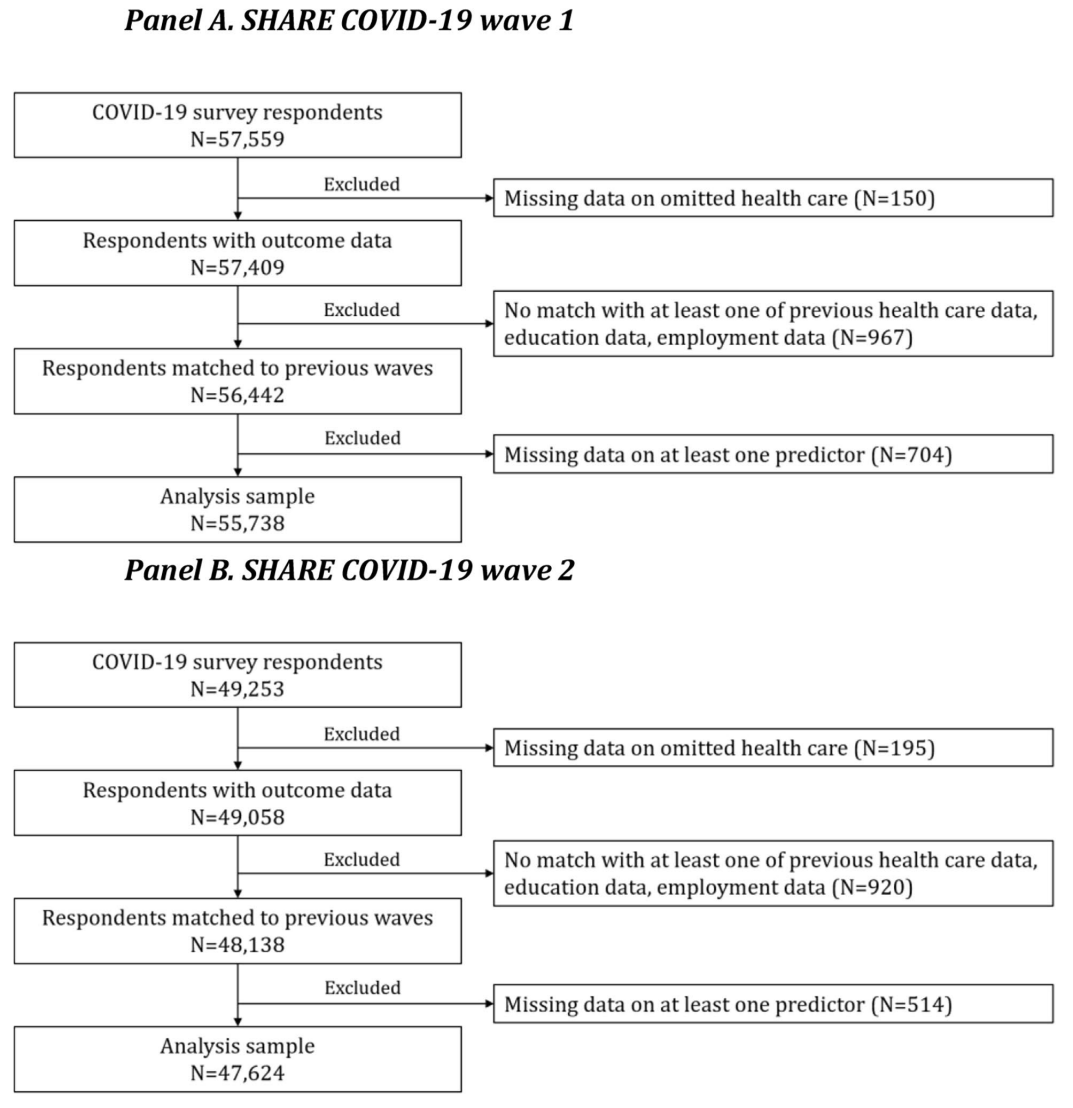
Sample selection process Note: From the analysis sample in Panel A, four individuals were dropped randomly to allow for five evenly sized folds for cross-validation.

This was a complete case analysis. Of the 57,559 individuals interviewed in the SHARE COVID-19 wave 1, we excluded 1,821 individuals (3% of the eligible sample) that had missing data on either the outcome or predictors (Fig. 1). This resulted in a sample of 55,738 individuals (97% of the eligible sample). We additionally excluded three randomly selected individuals to enable an even split into five samples for cross-validation procedures (more details below). Of the 49,253 individuals interviewed in the SHARE COVID-19 wave 2, we excluded 1,629 (3%) individuals due to missingness. All data preparation was conducted in Stata 17.

### Statistical analyses

We use four different algorithms to predict missed health care visits: stepwise selection (R package step), group lasso (R package grpreg), random forest (R package ranger), and neural networks (R package keras). This conceptually involved two steps. First, we identified which of the many available predictors should be included in the predictive models (known as feature selection). Second, we compared different ways of using these predictors – through different model types and algorithms – to predict missed visits.

Stepwise selection and group lasso approaches combine both feature selection and prediction. Since our outcome is a binary indicator of whether an individual missed a visit, both approaches used logistic regression models for prediction. For stepwise selection, the algorithm fits models of different sizes, sequentially deciding which predictor to add or drop to improve model performance. Among the many candidate models, the model with the highest Akaike Information Criteria (AIC) is chosen. For group lasso, a penalty on the number of predictors is added to the logistic regression optimization, such that predictors with minimal predictive power are excluded [21, 22].

Both stepwise and group lasso approaches are appealing because they are based around logistic regression models – and thus are straightforward to interpret – and because they provide a principled way of deciding which predictors to include. However, they are limited in that they require the analyst to specify the functional form of each predictor (e.g. should age be included as a linear, quadratic, or cubic function?) and which interactions between predictors should be included. Random forests and neural networks, in contrast, do not explicitly decide which predictors to include, but they do identify which interactions and functional forms maximize predictive power. Random forests are based on decision trees [23]. Decision trees are built by splitting the sample predictor by predictor such that the resulting sub-samples are more homogeneous regarding the outcome of interest. This results in a “tree”, where every split point (referred to as node) is essentially a decision rule on how to classify individuals based on the value of a predictor. We use the proportion of “positive” outcomes (i.e., missed health care visits) in a terminal subsample to estimate the probability of missed health care visits for a given individual (“probability tree”) [24]. Neural networks flexibly link predictors over several steps, known as “hidden” layers. In the simplest form (i.e., one “hidden” layer), linear functions with different weights for the predictors are combined to predict the outcome of interest. Then, the algorithm compares the predicted with the actual outcomes and adjusts the weights of the functions and the weights of the predictors within the functions to optimize the prediction.

The predictive power of group lasso, random forests and neural networks depend on the choice of specific parameters. To avoid overfitting the data and to ensure out-of-sample predictive validity, we choose the parameters using cross-validation. This involves splitting the data into several random subsets (known as folds), and using all folds but one to estimate the model. The model is then applied to the remaining fold to measure its out-of-sample performance. This procedure is repeated until every fold has been held out as a validation set. The specific characteristics we estimate through cross-validation are the penalty parameter for the group lasso, the optimal number of trees and the number of predictors among which the algorithm can choose to split the sample at each node for random forests, and the layer sizes and weights for neural networks. The final model parameters are depicted in the supplementary material.

Relatedly, the characteristics of each model depend on the input data, such that we assessed the performance of the final models using 5-fold cross-validation as well. All the models generate a predicted probability of missing a visit for each individual in the data (“risk score”). We average the predicted probability across all folds to assess the correlation between observed and predicted risk of missed health care visits. For a graphical representation, we calculate the average observed risk over percentiles of predicted risk scores in steps of 0.02.

We then assessed the predictive accuracy of the models when they are used not just to generate risk scores, but to classify individuals as at risk or not at risk. Whether the individual is classified as at risk of a missed visit depends on whether an individual’s risk score falls above or below a pre-specified probability cutoff. For all our results, we present the predictive performance measures across the entire range of potential probability cutoffs. This allows potential users to decide which cutoff meets their needs and resource constraints (e.g. a lower cutoff will likely have a higher true-negative rate but at the cost of resources needed to contact more individuals). Within each fold, we note the predictive accuracy (the proportion of correctly classified individuals), the sensitivity or true-positive rate (the proportion of individuals who were correctly classified among those who missed a care visit), and the specificity or true-negative rate (the proportion of individuals who were correctly classified among those who did not miss a care visit). We then graph these rates and average them over the five cross-validation folds for each algorithm.

An important question for predictive models is whether they will be accurate in different or new sources of data. While the cross-validation procedure improves this external predictive power, we additionally assessed the out-of-sample predictive accuracy of our model by applying it to data from the second round of the SHARE survey in 2021. Importantly, this second round of data was not used to build the models and thus this check helps us assess the external validity and long-term stability of our models. This is especially relevant from a policy perspective to evaluate how the models can be used in the future: A low stability over time would hint towards a spurious correlation of our current models with missed health care visits, such that they would need to be updated continuously to maintain their predictive power. In contrast, a high stability over time would indicate that our models capture some persistent, underlying patterns, increasing their applicability for targeting individuals at risk of missing care visits.

We run all analyses separated by sex to account for sex-specific trends. In addition, we examine algorithmic fairness with respect to age, education, and employment based on stratum-specific AUC as a measure of model performance. Lastly, we assess the importance of each predictor. For stepwise selection and group lasso, the measure of importance was the coefficient estimates from a logistic regression run on the full sample, including the predictors selected in the majority of the cross-validation folds. For random forest, we measured the importance of each predictor as the mean decrease in the Gini impurity.

All analyses were conducted in R 4.1.2.

## Results

### Sample characteristics

Table 1 displays the unweighted sample characteristics. There are more women than men in the sample (58% vs. 42%). Only a few respondents are younger than age 50 (< 1%); these respondents are partners of the main SHARE respondents. More than half of the respondents (56%) are between 60 and 74 years old. Correspondingly, most are retired (64%). More than two-thirds completed at least upper secondary education (71%).

Based on the previous diagnoses and medication, the majority of respondents require some form of regular care, especially for cardiovascular diseases such as hypertension (diagnosed: 56%, past medication: 56%) or high cholesterol (diagnosed: 41%, past medication: 37%). In the first wave, every sixth respondent reported some type of missed essential health care (16%). More specifically, 7% reported they forwent care due to fear of COVID-19, 10% that the medical staff postponed treatment due to COVID-19, and 2% that they were denied health care (multiple answers possible). In the second wave, this reduced to 9% of respondents with any type of missed essential health care, with 4% due to fear, 5% due to postponed treatment, and 2% due to denied health care.

**Table 1:** Sample characteristics

Outcome/predictor	Mean (SD)^a^	
	Wave 1	Wave 2
***Outcome***		
Any missed essential care	0.16 (0.36)	0.09 (0.29)
Forwent essential care	0.07 (0.25)	0.04 (0.19)
Postponed essential care	0.10 (0.30)	0.05 (0.22)
Denied essential care	0.02 (0.15)	0.02 (0.14)
***Sex***		
Female	0.58 (0.49)	0.58 (0.49)
Male	0.42 (0.49)	0.42 (0.49)
***Age*** ^***b***^		
Below 50	0.00 (0.07)	0.00 (0.06)
50–54	0.02 (0.13)	0.01 (0.10)
55–59	0.10 (0.29)	0.08 (0.27)
60–64	0.17 (0.37)	0.16 (0.37)
65–69	0.20 (0.40)	0.20 (0.40)
70–74	0.19 (0.39)	0.20 (0.40)
75–79	0.14 (0.35)	0.15 (0.36)
80–84	0.10 (0.31)	0.11 (0.31)
85–89	0.06 (0.23)	0.06 (0.23)
90+	0.03 (0.16)	0.03 (0.16)
***Education***		
None	0.03 (0.16)	0.03 (0.16)
Primary	0.15 (0.36)	0.14 (0.35)
Lower secondary	0.16 (0.37)	0.16 (0.37)
Upper secondary	0.37 (0.48)	0.38 (0.48)
Post-secondary	0.06 (0.23)	0.06 (0.23)
Tertiary	0.24 (0.43)	0.24 (0.43)
***Employment***		
Retired	0.64 (0.48)	0.64 (0.48)
Working	0.21 (0.41)	0.22 (0.41)
Unemployed	0.02 (0.14)	0.02 (0.14)
Disabled	0.03 (0.16)	0.03 (0.16)
Homemaker	0.08 (0.27)	0.08 (0.27)
Other	0.02 (0.12)	0.02 (0.12)
***Previous diagnoses*** ^***c***^		
None	0.40 (0.49)	0.41 (0.49)
Heart attack	0.20 (0.40)	0.20 (0.40)
High blood pressure or hypertension	0.56 (0.50)	0.56 (0.50)
High blood cholesterol	0.41 (0.49)	0.41 (0.49)
Stroke	0.07 (0.26)	0.07 (0.25)
Diabetes or high blood sugar	0.18 (0.38)	0.17 (0.38)
Chronic lung disease	0.10 (0.30)	0.10 (0.30)
Cancer	0.10 (0.30)	0.09 (0.29)
Stomach or duodenal ulcer, peptic ulcer	0.10 (0.30)	0.10 (0.30)
Parkinson disease	0.01 (0.11)	0.01 (0.10)
Cataracts	0.17 (0.38)	0.17 (0.37)
Hip fracture or femoral fracture	0.04 (0.19)	0.04 (0.19)
Other fractures	0.14 (0.35)	0.14 (0.34)
Alzheimer’s disease, dementia, senility	0.03 (0.17)	0.02 (0.16)
Other affective/emotional disorders	0.12 (0.32)	0.12 (0.32)
Rheumatoid arthritis	0.18 (0.38)	0.17 (0.38)
Osteoarthritis/other rheumatism	0.31 (0.46)	0.30 (0.46)
Other diagnosis	0.38 (0.49)	0.38 (0.48)
***Previous medication***		
Drugs for none	0.40 (0.49)	0.41 (0.49)
Drugs for high blood cholesterol	0.37 (0.48)	0.36 (0.48)
Drugs for high blood pressure	0.57 (0.50)	0.56 (0.50)
Drugs for coronary diseases	0.18 (0.38)	0.17 (0.38)
Drugs for other heart diseases	0.21 (0.41)	0.20 (0.40)
Drugs for diabetes	0.16 (0.37)	0.15 (0.36)
Drugs for joint pain	0.31 (0.46)	0.3ß (0.46)
Drugs for other pain	0.31 (0.46)	0.31 (0.46)
Drugs for sleep problems	0.15 (0.35)	0.14 (0.35)
Drugs for anxiety or depression	0.13 (0.33)	0.12 (0.33)
Drugs for osteoporosis (hormonal)	0.11 (0.32)	0.11 (0.32)
Drugs for stomach burns	0.17 (0.37)	0.16 (0.37)
Drugs for chronic bronchitis	0.05 (0.22)	0.05 (0.22)
Drugs for other	0.39 (0.49)	0.38 (0.49)

^a^Statistics are unweighted. ^b^Age is depicted as categories, but included as continuous predictor in the analyses. ^c^Multiple answers possible.

### Prediction accuracy

**Fig. 2:**
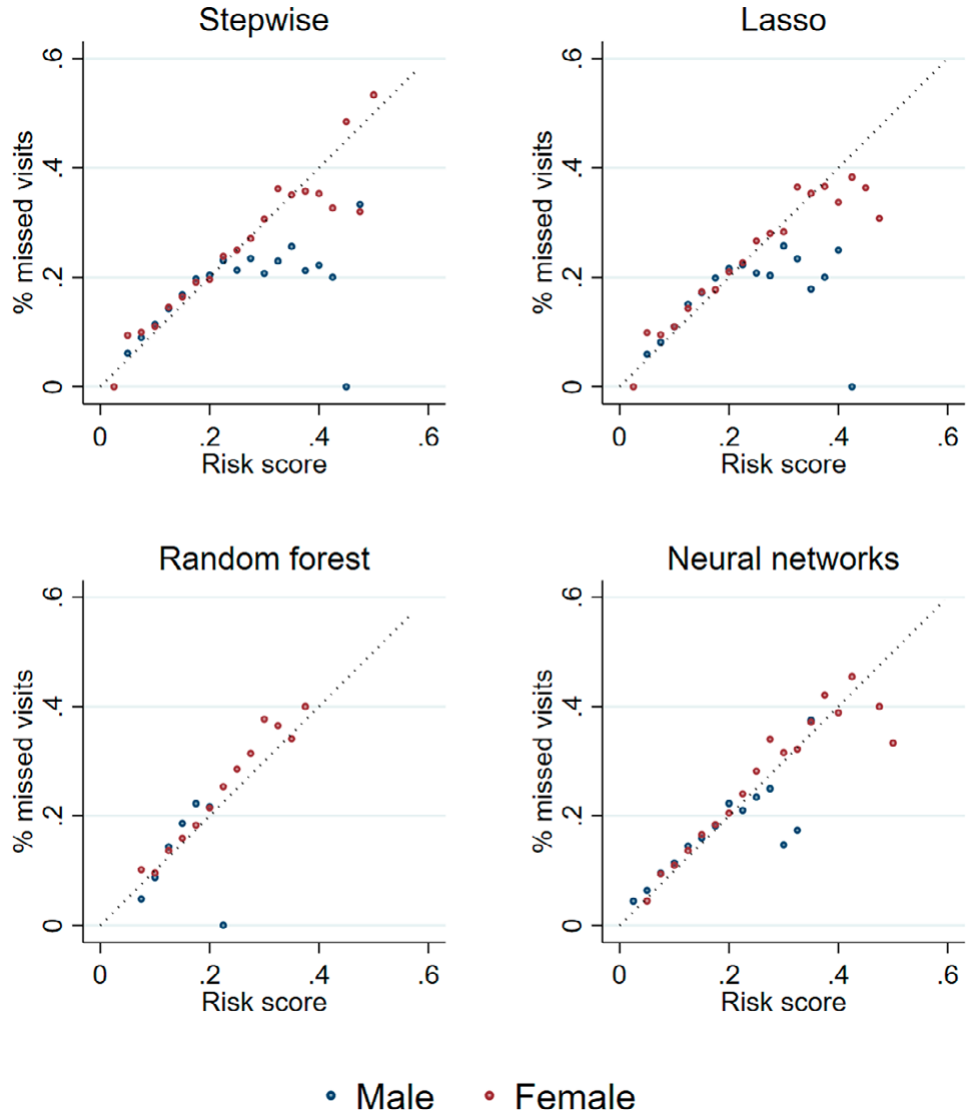
Predicted risk scores

Figure 2 depicts the share of missed visits by percentiles of the calculated missed visits risk score. Up to a risk score of approximately 0.2, the predicted risk score correlates well with the mean share of missed visits for all four methods and both sexes (all correlations are above 0.9 and significant at a level of 0.01). At higher risk scores, the correlation weakens especially for men, such that the correlation remains significant only for women and when using stepwise selection or group lasso. At the same time, the number of observations decreases strongly at higher risk scores, such that 80% of the sample have a risk score below 0.2, and more than 90% have a risk score below 0.25. Thus, for the majority of our sample, the risk score is highly predictive of the share of missed essential care visits.

**Fig. 3:**
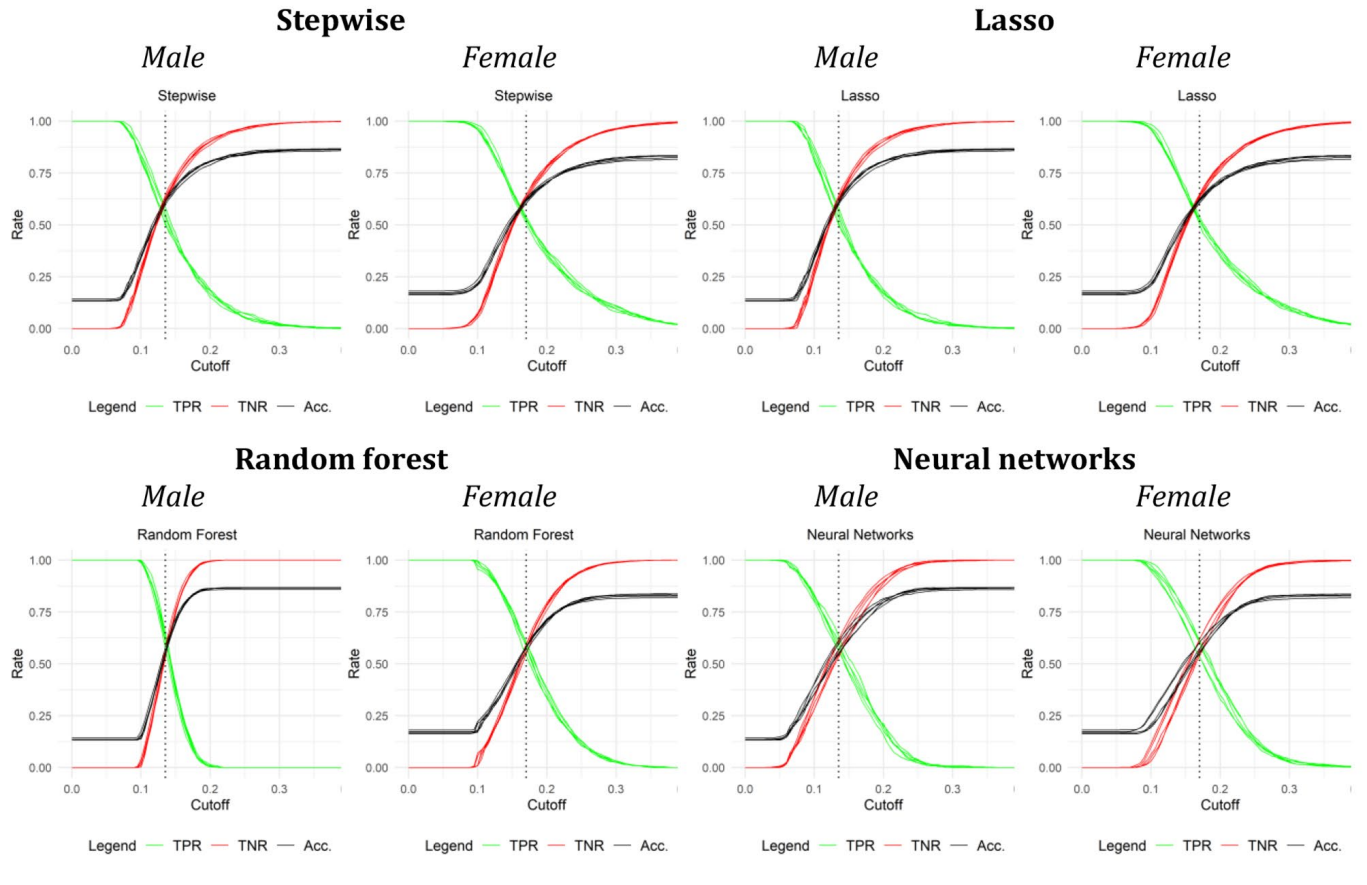
Performance measures Note: “TPR” is the true positive rate, “TNR” the true negative rate, and “Acc.” the accuracy. The outcomes for all five cross-validation steps are displayed. The cutoffs on the x-axis denote the predicted probability from which onwards individuals are labelled as “positive”, i.e. at risk of missing care

Figure 3 displays the predictive accuracy of the four machine learning approaches. Each graph shows three different measures: (1) the true positive rate (sensitivity) (2) true negative rate (specificity) and (3) accuracy. The x-axis for each graph shows the cutoff point that is used classify individuals with a risk score above this cutoff as individuals with a missed visit.

The accuracy of the predictions depends on which cutoff is used to classify individuals as at risk of missing a visit. Starting at a cutoff of approximately 0.08, the true positive rate starts to decrease and the true negative rate and the accuracy start to increase. This indicates that higher cutoffs increase accuracy by classifying a greater percentage of individuals as not at risk with the trade-off that fewer individuals that missed visits are correctly classified as at risk. The random forest algorithm produces much steeper curves than the other three methods, especially for men, with a sharp decline in the true positive rate and a sharp increase in the true negative rate and accuracy between cutoffs of 0.1 and 0.25. This indicates that the predicted probabilities of a missed health care visit mostly lie within this range and are thus more densely distributed compared to the other methods (Figure S1 in the supplementary material displays the predicted probabilities for each method and confirms this interpretation). Overall, this implies that for random forest, small changes in the cutoff come with relatively larger changes in the share of targeted individuals. The other three methods are less sensitive to changes in the cutoff.

To compare the performance of all four methods independent of the choice of cutoff, we display the area under the curve (AUC) in Table 2. This is the area under the curve when plotting the true positive rate on the false positive rate (1 - true negative rate). A method that would perform no better than chance would have an AUC of 0.5, an ideal method would have an AUC close to 1. Using this metric, all four methods perform very similarly with AUCs around 0.61. The results are qualitatively the same when disaggregating missed essential care visits into forgone visits due to fear, postponements by the medical staff, and denied care by the medical staff (Table S1 in the supplement).

**Table 2:** Performance measures

	Wave 1				Wave 2			
	Stepwise	Lasso	Random Forest	Neural Networks	Stepwise	Lasso	Random Forest	Neural Networks
	**Male**							
**AUC** **(SD)**	0.6084 (0.0148)	0.6130 (0.0136)	0.6150 (0.0134)	0.6120 (0.0113)	0.5857	0.5858	0.5923	0.5837
**Accuracy**	0.6104	0.6108	0.5531	0.5740	0.6137	0.6171	0.5425	0.5697
**True positive rate**	0.5364	0.5403	0.6171	0.5896	0.4943	0.4918	0.5844	0.5406
**True negative rate**	0.6220	0.6219	0.5432	0.5717	0.6240	0.6279	0.5389	0.5723
	**Female**							
**AUC** **(SD)**	0.6128 (0.0086)	0.6142 (0.0099)	0.6110 (0.0092)	0.6124 (0.0055)	0.6066	0.6061	0.6335	0.6031
**Accuracy**	0.6163	0.6177	0.5760	0.5778	0.6224	0.6202	0.6093	0.5761
**True positive rate**	0.5255	0.5242	0.5836	0.5767	0.5138	0.5134	0.5760	0.5685
**True negative rate**	0.6350	0.6369	0.5747	0.5785	0.6345	0.6321	0.6130	0.5770

Accuracy, true positive rate and true negative rate are assessed at a cutoff of 0.135 for men and 0.17 for women. Models were trained on data from wave 1 and then applied on data from wave 2. For wave 1, all measures are means across the five cross-validation folds for each method. For wave 2, the measures for stepwise and lasso are retrieved by applying the model from wave 1 on the data from wave 2. The measures for random forest and neural networks are retrieved by applying the models from all five cross-validation folds from wave 1 on the data from wave 2, and taking the mean predicted probability across all folds. (SD) is the standard deviation of the AUC across the five cross-validation folds for wave 1, and hence cannot be computed for wave 2.

We also display the accuracy, true positive rate, and true negative rate at a cutoff of 0.135 for men and 0.17 for women to exemplify the performance of the models (performance at further cutoffs is displayed in Table S2 in the supplement). At a cutoff of 0.135, between 54% (stepwise selection and lasso) and 62% (random forest) of the men missing care are correctly classified as at risk, while falsely targeting between 38% (stepwise and lasso) and 46% (random forest) of the men not at risk. For women, between 52% (lasso) and 58% (random forest) of the individuals at risk would be targeted using a cutoff of 0.17, at the expense of falsely targeting between 36% (lasso) and 43% (random forest) of the individuals not at risk. We choose different examples of cutoffs for men and women, as the distribution of risk scores is denser for men, such that at a risk score of 0.17, nearly all individuals would be labeled as not at risk (see Table S2 in the supplement). Also, women have on average a higher risk of a missed visit in our sample, resulting in a higher risk score. The optimal cutoff for targeting interventions depends on the trade-off the practitioner is willing to take between reaching out to the individuals at risk and falsely targeting individuals not at risk. As the distribution of predicted probabilities differs across the methods, the optimal cutoff also depends on the choice of the method.

Our models display consistent external validity and show similar prediction accuracies when fit to the second wave of the SHARE COVID 19 data. The AUC slightly drops from around 0.61 to around 0.59 for men and stays nearly stable for women (from around 0.61 for all methods to 0.61 for stepwise selection and lasso, 0.63 for random forest, and 0.60 for neural networks). Similarly, the accuracy and the true negative rate stay quite stable, while the true positive rate decreases slightly, especially for men (approx. 4% points across models).

### Coefficient estimates

We compare the coefficient estimates of the two feature selection algorithms in Figure S3 in the supplement. Both algorithms retain most of the predictors, with the stepwise selection dropping slightly more predictors than the group lasso. For both algorithms, more predictors are retained for the female than the male subsample. Being disabled is a consistent and large predictor of missed health care visits across the models, which is in line with findings from the US [25]. Other important predictors are previous osteoarthritis/other rheumatism, emotional disorders, cataracts, or cancer diagnosis, and medication for joint pain, anxiety/depression, or stomach burns. Alzheimer’s disease is consistently correlated with a lower risk of missed health care visits. This partly mirrors findings from a survey based on Medicare beneficiaries in the US [26].

### Performance by sociodemographic group

**Fig. 4:**
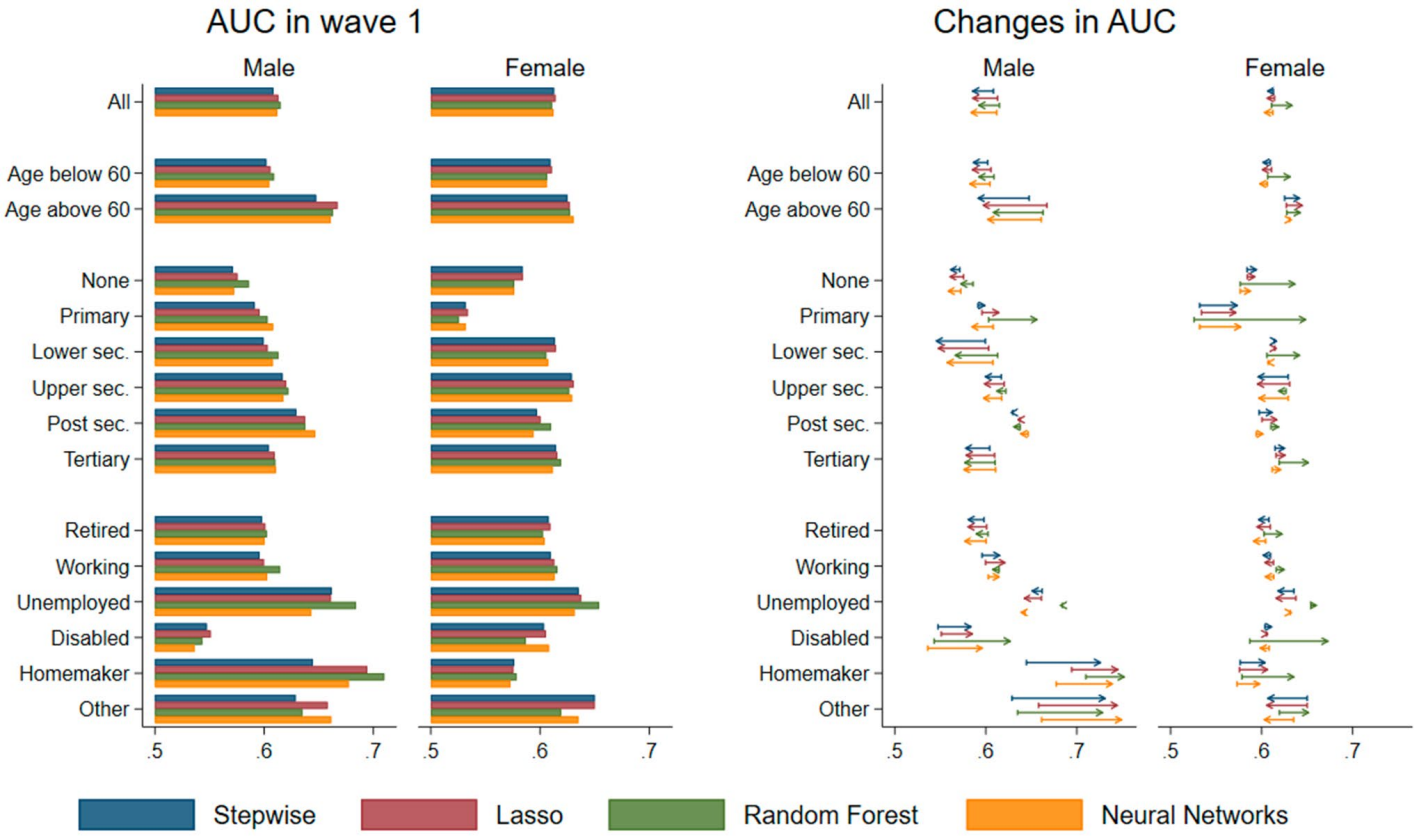
AUC by sociodemographic group

We analyze the algorithmic fairness of our models by comparing the AUC for different sociodemographic groups. As depicted in Fig. 4, the AUC in wave 1 is lower for individuals younger than 60 and with lower levels of education. It is particularly low for male disabled respondents and female respondents with primary education. The strongest differences between the models emerge for the different occupational groups. For example, compared to the other models, the random forest model is relatively effective at predicting which individuals are at risk of a missed care visits for unemployed individuals (irrespective of sex), and men who are working or are homemakers. At the same time, it is relatively ineffective at predicting missed care visits for individuals who are disabled or categorized as “other”.

In wave 2, the differences in AUC by age are diminished for men, but slightly increased for women. For men, the decreases in AUC occur especially for individuals with lower secondary or tertiary education, while for those who are disabled, homemakers or have some other occupational status, the AUC strongly increased. For women, the AUC increased particularly for individuals with primary education and those who are homeworkers, especially when considering the random forest algorithm.

## Discussion

We show that machine learning algorithms based on few common patient characteristics can be used to predict which individuals are most likely to miss essential health care visits during disruptions to health care such as the COVID-19 pandemic. Importantly, these models all perform better than random chance. We also find that simple feature selection algorithms (stepwise selection and group lasso) perform similar to more complex algorithms (random forest and neural networks). This means that health care providers or insurance companies do not need high computing power or specialized staff to implement our models in real-world practice. Importantly, our models retain their performance over time and consistently predict missed health care visits when applied to new data collected one year after the data used to build our models: Our models may thus be applicable beyond COVID-19 and be used to reduce missed visits during future disruptions to health care services.

Previous studies demonstrated that machine learning algorithms can be powerful tools to predict health care visits or no-shows [27–32]. But their applicability in practice depends on the availability of predictors and the measurability of the outcome of interest: For example, while data from electronic health records might contain a large number of predictors, including medical findings, health insurance providers might have access to claims data only [33]. Still, a recent study showed that administrative data alone can be a powerful predictor for health care visits, with little additional improvement when adding further anthropometric data or information on neighborhood socioeconomic status [28]. Similarly, non-attendance of appointments can be predicted very well based on previous appointments [30–32], but this data might not be available to health insurance providers. Moreover, even if such data is available, decisions to not visit a facility will not be recorded in administrative or clinical data. Our approach bridges this gap by using survey data, albeit at the costs of having fewer and more coarse predictors at hand, resulting in a lower predictive power than reached by studies on health care visits or missed appointments. More specifically, our models reach an AUC of approximately 0.6 across specifications and outcomes, while studies predicting missed appointments or health care visits often reach an AUC of approximately 0.8 [27–31]. While most of them rely on detailed data on previously missed appointments or electronic health records, the study perhaps closest to ours uses administrative records (though including a predicted cost score) to predict health care visits, and reaches an AUC between 0.78 and 0.84, depending on the outcome [28]. Thus, although the SHARE data already includes a comprehensive set of diagnoses and medication, future research could investigate whether extending these categories further would improve the predictive power of models for missed health care visits.

The temporal stability of our predictions increases our confidence in the external validity of our models and their applicability beyond the first one and a half years of the pandemic. This is particularly important as, in contrast to previous pandemics such as the Severe Acute Respiratory Syndrome (SARS) or the Middle East Respiratory Syndrome (MERS) pandemics, COVID-19 is characterized by a longer duration with multiple waves. Thus, while the utilization of health care services recovered quickly in the case of SARS and MERS [34, 35], current evidence indicates a slow recovery and repeated disruption of health care visits during COVID-19 [1, 3, 6]. The relentless nature of the COVID-19 thus carries greater risks than prior pandemics. Individuals need to be contacted and transferred back to the health care systems before they chronically forgo essential care or delay necessary care so long that they increase their risk of severe health complications.

Our predictions can be directly used to improve the targeting of efforts to prevent individuals aged 50 or older from missing health care visits in the European Union (except Ireland), Switzerland, and Israel. We will publicly post the required weights and model parameters online, such that health insurance providers or similar stakeholders can estimate the probabilities of missed health care visits for their clients. Depending on the intended intervention, stakeholders can choose the cutoff which yields their preferred trade-off between reaching out to clients at risk, and falsely targeting clients not at risk. Moreover, as our analysis does not assess the potential negative consequences of missed health care visits, stakeholders might choose to weight our predictions of missed health care visits with their own assessments of severity of missed health care visits for specific subgroups, such as clients with a chronic illness.

Our study faces several limitations. First of all, our data relies on self-reported information on missed essential health care visits. This might introduce a bias if discrepancies between self-reported and objective missed health care visits are not random. In addition, participants were asked whether they had missed any health care visits since the onset of the COVID-19 pandemic. This might imply different recall periods for participants, as the onset of the pandemic is not a clear date and varies across locations. Still, given that nearly all European countries imposed the first lockdown within a time span of two weeks [36], and that this lockdown was an unparalleled, significant event, the resulting bias might be low. Similarly, most of the interviews took place within two months. While this might increase the recall period for the later participants, the interviews were conducted between the first and the second COVID-19 wave, in a time of comparatively low infection and death rates, such that most of the missed health care visits are expected to have already taken place before the start of the survey. Also, we do not include missed health care at specialists, as the data combines specialists and dentists in one item. Given the high share of missed regular dentist check-ups recorded in other studies [37, 38], we expect that these contribute to the majority of missed visits in this item, and thus are confident that excluding it leads to a more accurate identification of missed essential health care services. Finally, with the panel structure of the data and the fact that our predictors stem from previous waves, i.e., except for age did not change between the two COVID-19 surveys, we might ask whether the stability in performance across both ways might be driven by some form of path dependence: The same individuals who reported missed visits in the first wave might report missed visits in the second wave as well. However, a closer look at the data reveals some movement between the waves: Only about a fifth of the individuals with missed care in wave 1 reported a missed care visit in wave 2, while about 7% of the individuals without missed care in wave 1 reported a missed care visit in wave 2. As well, individuals with missed visits in both waves have a higher risk score on average than individuals without any missed visits or a missed visit in wave 1 only, as depicted in Figure S2 in the supplement. This indicates that the models pick up some underlying pattern in the predictors which correlates with the risk of missing care in both waves, rather than the risk in wave 1 alone. Yet, as the stability of the model predictions varies across sociodemographic groups, there are possible gaps in targeting across these groups when applying the models.

## Conclusions

The COVID-19 pandemic put health systems worldwide under pressure and led to severe disruptions in health care. With the support of machine learning methods, routinely-collected survey data can be used to target individuals at risk of missed health care more efficiently during periods of severe and prolonged health care disruption.

## Electronic supplementary material

Below is the link to the electronic supplementary material.

Supplementary Material 1

## Data Availability

The datasets analysed during the current study are available in the SHARE repository (10.6103/SHARE.w1.800, 10.6103/SHARE.w2.800, 10.6103/SHARE.w3.800, 10.6103/SHARE.w4.800, 10.6103/SHARE.w5.800, 10.6103/SHARE.w6.800, 10.6103/SHARE.w7.800, 10.6103/SHARE.w8.800, 10.6103/SHARE.w8ca.800, 10.6103/SHARE.w9ca.800). Access to the SHARE data can be requested at the SHARE website (https://share-eric.eu/data/data-access) and is free of charge for scientific purposes. Code can be requested from the authors and will be made freely available upon publication of the study at https://osf.io/tqdk9/.
